# Charge and Peptide Concentration as Determinants of the Hydrogel Internal Aqueous Environment

**DOI:** 10.3390/ma12050832

**Published:** 2019-03-12

**Authors:** Scott V. Elgersma, Michelle Ha, Jung-Lynn Jonathan Yang, Vladimir K. Michaelis, Larry D. Unsworth

**Affiliations:** 1 Department of Chemical and Materials Engineering, Faculty of Engineering, University of Alberta, Edmonton, AB T6G 1H9, Canada; selgersm@ualberta.ca (S.V.E.); junglynn@ualberta.ca (J.-L.J.Y.); 2 National Research Council, National Institute for Nanotechnology, Edmonton, AB T6G 2M9, Canada; 3 Department of Chemistry, Faculty of Science, University of Alberta, Edmonton, AB T6G 2G2, Canada; mha1@ualberta.ca (M.H.); vmichael@ualberta.ca (V.K.M.)

**Keywords:** self-assembly, peptide chemistry, (RADA)_4_, vicinal water structure, nanofiber, hydrogel

## Abstract

Self-assembling peptides are a promising class of biomaterials with desirable biocompatibility and versatility. In particular, the oligopeptide (RADA)_4_, consisting of arginine (R), alanine (A), and aspartic acid (D), self-assembles into nanofibers that develop into a three-dimensional hydrogel of up to 99.5% (w/v) water; yet, the organization of water within the hydrogel matrix is poorly understood. Importantly, peptide concentration and polarity are hypothesized to control the internal water structure. Using variable temperature deuterium solid-state nuclear magnetic resonance (^2^H NMR) spectroscopy, we measured the amount of bound water in (RADA)_4_-based hydrogels, quantified as the non-frozen water content. To investigate how peptide polarity affects water structure, five lysine (K) moieties were appended to (RADA)_4_ to generate (RADA)_4_K_5_. Hydrogels at 1 and 5% total peptide concentration were prepared from a 75:25 (w/w) blend of (RADA)_4_:(RADA)_4_K_5_ and similarly analyzed by ^2^H NMR. Interestingly, at 5% peptide concentration, there was lower mobile water content in the lysinated versus the pristine (RADA)_4_ hydrogel. Regardless of the presence of lysine, the 5% peptide concentration had higher non-frozen water content at temperatures as low as 217 ± 1.0 K, suggesting that bound water increases with peptide concentration. The bound water, though non-frozen, may be strongly bound to the charged lysine moiety to appear as immobilized water. Further understanding of the factors controlling water structure within hydrogels is important for tuning the transport properties of bioactive solutes in the hydrogel matrix when designing for biomedical applications.

## 1. Introduction

Self-assembling peptide hydrogels offer a wide range of potential biomedical applications, ranging from drug delivery platforms [1,2], fillers for treating osteoarthritis [3], engineered matrices for neuronal cultures [4,5,6,7], to hemostatic agents [8]. The generally non-immunogenic, non-inflammatory, and non-thrombogenic properties [8,9] make self-assembled peptides compatible for these applications. In particular, hydrogels prepared from (RADA)_4_ possess desirable physicochemical and biocompatible properties [10,11,12]. (RADA)_4_ consists of four tetrapeptide repeats containing arginine (R), alanine (A), and aspartic acid (D), which self-assemble into nanofibers [4] in physiologically relevant aqueous solution. The biocompatible nanofibers are simple to synthesize and form a hydrogel of net neutral charge that can respond to external stimuli at physiological conditions [13,14,15,16]. (RADA)_4_ has the potential for minimally invasive therapies (i.e., injectable), protein delivery, and sustaining three-dimensional cellular activities [10,15]. However, the limited understanding of the peptide self-assembly process at the molecular level impedes the practical application of hydrogel biomaterials.

(RADA)_4_ hydrogels are intensely hydrated, up to 99.5% (w/v) water [7,13,15], and the organization of water controls the transport properties within hydrogel matrices [17]. At the molecular level, three distinct phases of water are thought to exist within hydrated polymers: bulk water, vicinal (loosely or freezing bound) water, and strongly (non-freezing) bound water [17,18,19,20,21,22,23,24,25]. Bulk water has little to no interaction with the polymer and crystallizes at 0 °C. Vicinal water interacts with the hydrophilic moieties of the polymer and freezes below 0 °C. In contrast, due to substantial intermolecular interactions with the polymer, strongly bound water may remain mobile at temperatures as low as −100 °C [26]. Of particular significance are the bound water shells that solvate the peptide nanofibers of the hydrogel. These layers of hydration may lead to an increase in the viscosity experienced by diffusing molecules within the hydrogel and thus affect macroscopic release kinetics, as illustrated by the Stokes–Einstein equation. Thus, by controlling the hydrogel’s internal water structure, it may be possible to alter the diffusion rate for releasing absorbed solutes from the hydrogel.

Herein, we explored peptide concentration and charge as factors that influence the organization of water in (RADA)_4_-based hydrogels. Increased peptide concentration led to a higher amount of non-frozen water. Interestingly, the incorporation of charged lysine groups to (RADA)_4_ reduced the mobile water content, which may have been due to the enhanced peptide–water binding that hindered the motion of water molecules. These data provide novel insight on the molecular basis of the aqueous environment in peptide hydrogels.

## 2. Materials and Methods

### 2.1. Peptide Synthesis

The peptides acetyl-[Arg-Ala-Asp-Ala]_4_-CONH_2_, denoted as (RADA)_4_, and acetyl-[Arg-Ala-Asp-Ala]_4_-[Lys]_5_-CONH_2_, denoted as (RADA)_4_K_5_, were commercially synthesized and purified (RS Synthesis, Louisville, Kentucky, USA). The molecular weights of 1711.8 Da for (RADA)_4_ and 2353.3 Da for (RADA)_4_K_5_, determined by the ABI 4800 matrix-assisted laser desorption/ionization time of flight mass spectroscopy (Applied Biosystems/MDS SciEx, Concord, Ontario, Canada), were as expected compared to the estimated molecular weights of 1712.8 and 2353.7 Da, respectively. Sample purity, ~90% purity for both peptides, was analyzed by high performance liquid chromatography (Agilent Technologies, Santa Clara, CA, USA) analysis using a Luna C18 reverse phase 4.6 × 250 mm column. The spectra were quantified on the Agilent Technologies in-house software.

### 2.2. Hydrogel Preparation

Hydrogels were prepared according to established procedures [25]. Lyophilized (RADA)_4_ peptide (RS Synthesis, Louisville, Kentucky, USA) was dissolved in 1× phosphate buffered saline (PBS, pH 7.4, Sigma-Aldrich, St. Louis, Missouri, USA) with D_2_O as the solvent for ^2^H NMR experiments. The peptide solution was sonicated (2800 Branson sonicator, Crystal Electronics, Newmarket, Canada) at 25 °C for 30 min and diluted with PBS to obtain final peptide concentrations of 1 and 5% (w/v) (RADA)_4_, denoted 1K0 and 5K0, respectively. The diluted peptide was allowed to self-assemble into hydrogel at 37 °C for at least 1 h. To prepare blended (RADA)_4_–(RADA)_4_K_5_ hydrogels, (RADA)_4_K_5_ (RS Synthesis, Louisville, Kentucky, USA) was similarly dissolved in PBS, sonicated, and combined with (RADA)_4_ solution for self-assembly. Hydrogels containing a 75:25 (w/w) ratio of (RADA)_4_:(RADA)_4_K_5_ were prepared at 1 and 5% total peptide concentrations, denoted 1K25 and 5K25, respectively. 

### 2.3. Atomic Force Microscopy

Atomic force microscopy (AFM) was conducted to confirm nanofiber formation in peptide solutions [27]. 1K0 and 1K25 solutions at 0.01% (w/v) were prepared in 10 mM phosphate buffer, pH 7.4. Five microlitres of each solution was placed on a freshly cleaved muscovite mica substrate (V-1 quality; Emsdiasum, Hatfield, Pennsylvania, USA) and dried in a dessicator for 3 h minimum. A NanoWizard II atomic force microscope (JPK Instruments, Berlin, Germany) equipped with a cantilever was used in intermittent mode to probe samples. The 240 µm long silicon cantilever probe with aluminum reflex coating had a resonance frequency of 50–90 kHz, spring constant of 0.7–3.8 N/m, and tip radius of ≤7 nm (Olympus AC240TS, Tokyo, Japan). AFM images were collected from 5 different areas for each sample from 3 independent experiments and analyzed using the Gwyddion 2.2 software (Czech Metrology Institute, Brno, Czech Republic) to quantify nanofiber length and width.

### 2.4. Solid–State ^2^H NMR

Solid–state ^2^H NMR experiments were performed at 11.75 T (*v*_0_ = 76.8 MHz) on a Bruker Avance 500 spectrometer using a Bruker 4 mm double resonance probe. Samples were packed in zirconia rotors (4 mm outer diameter) and acquired under non-spinning conditions. ^2^H NMR spectra were obtained with a solid-echo pulse sequence with 5.0 μs 90° pulses, a 30.0 μs interpulse delay (τ_1_), a 60 ms acquisition time, and a 2.0 s recycle delay. Data were left-shifted to ensure the free induction decays began at the maximum. Variable-temperature NMR data were obtained on the Avance 500 NMR spectrometer using a BVT3000 variable temperature unit. The heat exchanger source was an ethanol/dry ice bath, with dry air as the VT gas. Gas flow rates of 1070 and 1470 L/h were used to reach the target temperatures. Temperature calibrations were performed according to established methods using methylammonium lead chloride ^207^Pb chemical shifts [28]. The NMR spectra were simulated using the WSolids NMR Simulation Package (Version 1.21.3; Universität Tübingen, Tübingen, Germany) to quantify the mobile water in hydrogel samples [29]. 

## 3. Results and Discussion

Analyzing the water structure in the peptide matrix is fundamental to understanding the internal transport properties of hydrogels for biomedical applications. Within the matrix, liquid water is partitioned into bulk and bound phases. The bound phase, in particular, belongs to the solvation shell, where water molecules are in close proximity, or directly hydrogen bonded, to the peptide nanofibers. It is unclear how the relative amount of bulk versus bound water is affected by polar moieties in the peptide. As such, we appended five lysine units to the C-terminus of (RADA)_4_ to generate (RADA)_4_K_5_. Lysine is positively [30] charged in pH 7.4 solution with a hydration potential [31] of −9.52 kcal/mol at pH 7 and thus expected to increase the hydrophilicity of pristine (RADA)_4_. The compact epsilon ammonium group of lysine, in particular, has a high charge concentration and is expected to bind water more strongly than other hydrophilic groups, such as the guanidinium group of arginine, where the charge is dispersed over the entire guanidinium framework [32]. Furthermore, peptide self-assembly into nanofibers is a pre-requisite for hydrogel formation. Pristine (RADA)_4_ can self-assemble into nanofibers but pure (RADA)_4_K_5_ cannot [12,33]. Strong interactions between water and the charged lysine groups and/or electrostatic repulsion may prevent nanofiber formation [33]. Self-assembly is possible, however, by blending (RADA)_4_ with up to 25% (RADA)_4_K_5_ [11]. 

**Figure 1:**
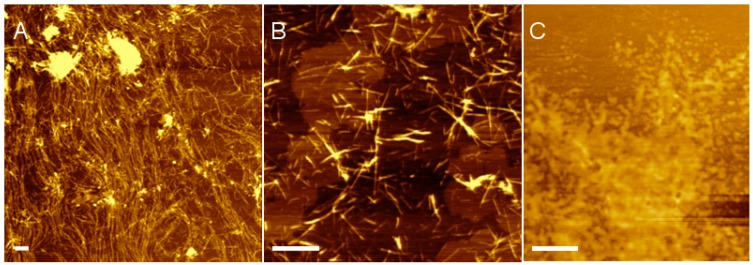
Atomic force microscopy (AFM) images of nanofiber morphology in (**A**) pristine (RADA)_4_, (**B**) a blend of 75%(RADA)_4_:25%(RADA)_4_K_5_ (w/w), and (**C**) (RADA)_4_K_5_. Peptide solutions were prepared at 0.01% (w/v) in 10 mM phosphate buffer, pH 7.4. Scale bars 500 nm.

To confirm nanofiber self-assembly, hydrogels prepared from pristine (RADA)_4_ and the blend of 75:25 (RADA)_4_:(RADA)_4_K_5_ were analyzed by atomic force microscopy (AFM). It was unnecessary to analyze both the 1 and 5% peptide concentrations since all AFM samples were significantly diluted, and the final concentration would be equal regardless of the initial peptide concentration in the hydrogel. Figure 1 shows AFM images of nanofiber morphology at 0.01% (w/v) total peptide concentration in 10 mM phosphate buffer, pH 7.4. Indeed, both pristine (RADA)_4_ (Figure 1A) and the lysinated blend (Figure 1B) self-assembled into nanofibers. As expected, (RADA)_4_K_5_ alone did not form nanofibers but collapsed into random aggregates (Figure 1C). The length of pristine (RADA)_4_ nanofibers ranged from hundreds of nanometers to several micrometres (sampled across 5 areas and 3 independent experiments). The 10.3 ± 1.5 nm width and 1.3 ± 0.3 nm height of nanofibers were consistent with previously reported dimensions [34]. In contrast, the nanofibers of the 75:25 (RADA)_4_:(RADA)_4_K_5_ blend were 347 ± 201 nm in length, though not statistically shorter than those of pristine (RADA)_4_, and comparable in width (17.7 ± 3.3 nm) and height (1.4 ± 0.2 nm). Therefore, while both the pristine and lysinated blend of (RADA)_4_ could self-assemble into nanofibers, the lysine moieties in (RADA)_4_K_5_ may limit nanofiber length.

Differential scanning calorimetry (DSC) was previously used to examine the organization of water in (RADA)_4_ hydrogel, specifically the relative amount of freezing versus non-freezing water [25]. DSC measures the heat transfer associated with freezing to quantify the amount of frozen water in the bulk phase. Subtracting the frozen from the total water gives the difference as the non-frozen water content, consisting of vicinal and strongly bound water [35]. In contrast, whereas DSC indirectly quantifies bound water, with deuterium nuclear magnetic resonance (^2^H NMR) spectroscopy one may directly assess bound water content based on the ^2^H relaxation characteristics of D_2_O and through an analysis of the ^2^H NMR line shapes [35,36,37,38]. Through variable temperature NMR, the water environment can be identified by the temperature at which the water molecules become mobile and thus alter the resulting NMR lineshape: 170–190 K (strongly bound water), 230–260 K (vicinal water), and ~273 K (bulk water) [26,35,39,40,41]. Since DSC becomes insensitive to changes in thermal energy below 200–230 K [35], NMR is more appropriate than DSC to distinguish vicinal water from strongly bound water at low temperatures and to provide a direct overall assessment of water structure.

**Figure 2:**
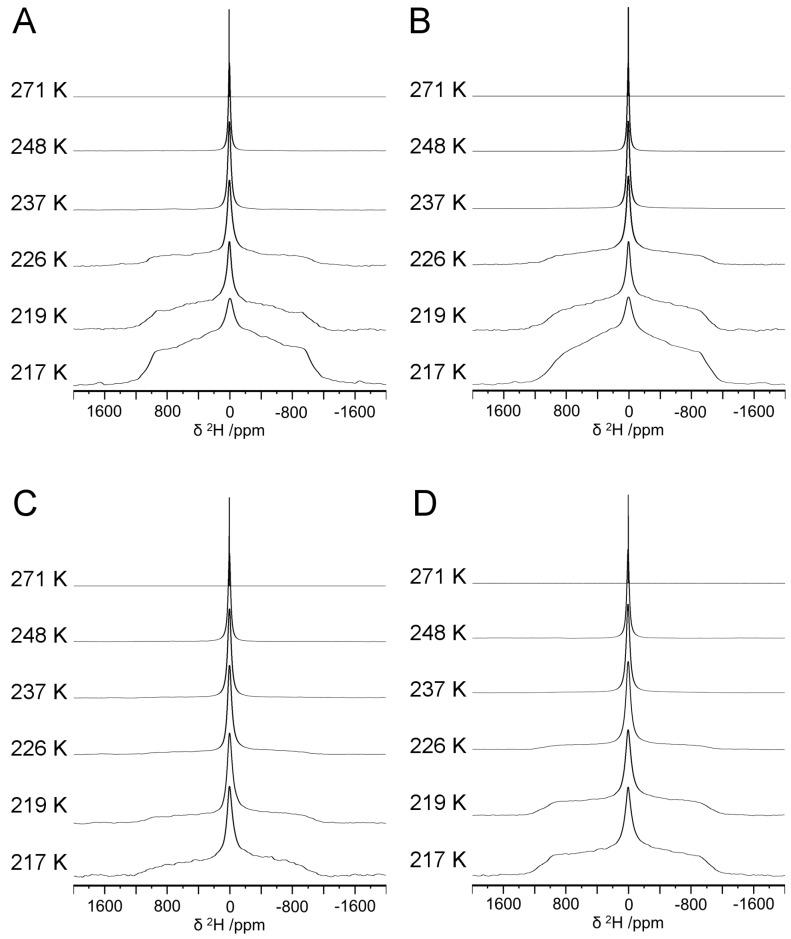
Variable temperature ^2^H NMR spectra for (RADA)_4_-based hydrogels formulated as (**A**) 1K0, (**B**) 1K25, (**C**) 5K0, and (**D**) 5K25. All spectra were acquired at 11.75 T (*v*_0_ = 76.8 MHz).

To study the effects of peptide concentration and charge on internal water structure, hydrogels were prepared at 1 and 5% (w/v) total peptide concentration, and five lysine moieties were appended to (RADA)_4_ to generate (RADA)_4_K_5_. At 1%, hydrogels were prepared from pristine (RADA)_4_ (1K0) and the blend of 75:25 (RADA)_4_:(RADA)_4_K_5_ (1K25). At 5%, hydrogels were similarly prepared from pristine (RADA)_4_ (5K0) and the lysinated blend (5K25). The hydrogels were analyzed by variable temperature ^2^H NMR from 271 to 217 ± 1.0 K (Figure 2). The single narrow peak (~0 ppm) at 271 ± 1.0 K observed for all samples indicates the fast, isotropic tumbling of water molecules in the liquid phase such that the mobility of water molecules was not constrained relative to the NMR timescale. As the motion of water molecules was constrained upon cooling, the base of the central peak broadened into roughly symmetrical shoulders. At 226 ± 1.0 K, a broad shoulder appeared for 1K0 (Figure 2A) and 1K25 (Figure 2B). These shoulders increased in relative intensities as the temperature was further reduced to 217 ± 1.0 K, demonstrating that the motion of water molecules became restricted. However, the broad powder pattern (Pake doublet [42]) characteristic of hexagonal D_2_O ice (i.e., frozen non-mobile crystalline water) was absent at 217 ± 1.0 K, well below the freezing point of pure D_2_O (277 K) [43]. This demonstrated that the motion of water molecules became increasingly hindered below 226 ± 1.0 K, but not necessarily locked in a crystalline state. The ^2^H NMR lineshape is consistent with water exhibiting twofold hopping motion. A similar peak broadening occurred for the spectra of 5K0 (Figure 2C) and 5K25 (Figure 2D) hydrogels as the temperature was lowered. However, the shoulders were subtle at 226 ± 1.0 K for the 5 compared to the 1% hydrogels, suggesting that the increased mobility of water molecules was maintained to a lower temperature in 5 compared to 1% hydrogels.

**Figure 3:**
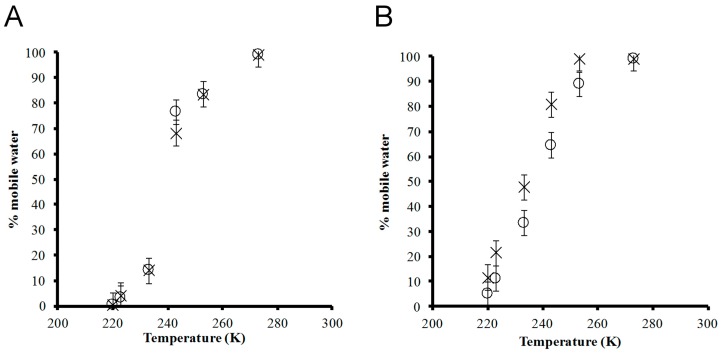
Non-spinning ^2^H NMR spectra were used to quantify (peak areas) the relative mobile water content as a function of temperature in the hydrogels (**A**) × 1K0 and ○ 1K25 and (**B**) × 5K0 and ○ 5K25. The mobile water content is expressed as a percentage of the initial water content at room temperature.

The relative quantity of mobile water molecules in each hydrogel was obtained by fitting the NMR spectra using WSolids, where two distinct sites (mobile vs. restricted water) were simulated with 50:50 Gaussian:Lorentzian line broadening. Figure 3 illustrates the percentage of mobile water, relative to the initial water content at room temperature, in each hydrogel sample from 271 to 217 ± 1.0 K. At 1% peptide concentration, the curves for 1K0 and 1K25 almost overlap (Figure 3A). At 271 ± 1.0 K, virtually all water (~99%) was mobile, consistent with the single central peak observed for all ^2^H NMR spectra (Figure 2). The amount of mobile water decreased with temperature. There is a sharp decrease between 237 and 226 ± 1.0 K, where the amount of mobile water dropped by 54 and 63 ± 5% for 1K0 and 1K25, respectively. Notably, there was similar non-frozen water content (16 ± 1%) in 1K0 analyzed by DSC [25] compared to the mobile water content (14 ± 5%) measured by NMR at 226 ± 1.0 K, which may be interpreted as the bound water content of the hydrogel. DSC operates at a temperature where the vicinal and strongly bound water are not necessarily frozen, and thus, this method can detect the frozen bulk water but cannot differentiate the two non-frozen phases. The non-frozen water content measured by DSC is expected to be greater than or equal to the mobile water content measured by NMR. NMR, conducted at temperatures over which vicinal water freezes [35,39], can further distinguish the vicinal from the strongly bound water. The lysinated and pristine (RADA)_4_ had the greatest difference in mobile water content within 230–260 K, the freezing range of loosely bound vicinal water [35]. At 237 ± 1.0 K, the higher mobile water content of lysinated (RADA)_4_ agrees with previous DSC analysis in which a 0.5% sample of the lysinated blend had higher non-frozen content water than pristine (RADA)_4_ (14.86% in the lysinated blend versus 8.51% in (RADA)_4_) [11]. Upon further cooling to 217 ± 1.0 K, the NMR data showed that the amount of mobile water further decreased to 0.1 ± 5% for both 1K0 and 1K25. Thus, the incorporation of lysine appeared to improve water–peptide binding to the extent of increasing the vicinal water content.

At 5% peptide concentration, pristine (RADA)_4_ contained a greater amount of mobile water than the lysinated blend at decreasing temperatures (Figure 3B). The temperature dependence of the mobile water content showed a similar sigmoid behaviour as in 1% peptide hydrogels. Nearly all the water content was mobile at 271 ± 1.0 K. A slight decrease in mobile water occurred at 248 ± 1.0 K for 5K25, but almost all water remained mobile in 5K0 at this temperature. Upon cooling to 217 ± 1.0 K, the amount of mobile water dropped monotonically to 12 and 5 ± 5% for 5K0 and 5K25, respectively. Over the observed temperature range, there was generally more mobile water in the 5 than the 1% hydrogel, which agrees with the DSC data such that increased peptide concentration (from 0.5 to 3.0% (RADA)_4_) leads to less frozen water (from 99.02 to 21%) and more non-frozen water (from 8.5% to 77%) [25]. Potentially, the nanofiber content increases along with its associated surface area available to interact with water [25].

Unexpectedly, the mobile water content of the lysinated blend was generally below that of pristine (RADA)_4_. In contrast, previous analysis by DSC indicated that, in a 0.5% hydrogel, the lysinated blend decreased frozen water content (90.19% in (RADA)_4_ versus 82.31% in the lysinated blend) and increased non-frozen water content (8.51% in (RADA)_4_ versus 14.86% in the lysinated blend) [11]. Specific to the 5% hydrogel, an explanation as to why appending a charged group, such as lysine, would yield an apparent lower amount of mobile water is that the increased hydrophilicity, together with a higher peptide concentration, results in a stronger, as well as larger solvation cage than pristine (RADA)_4_ or the 1% lysinated blend. The tightly bound water molecules in the 5% lysinated blend may not undergo sufficient molecular motion to be deemed mobile by NMR. In support, as the temperature was lowered, the decrease in mobile water was not met with increased NMR signal for crystalline water, demonstrating that the immobilized water was not necessarily frozen.

**Figure 4:**
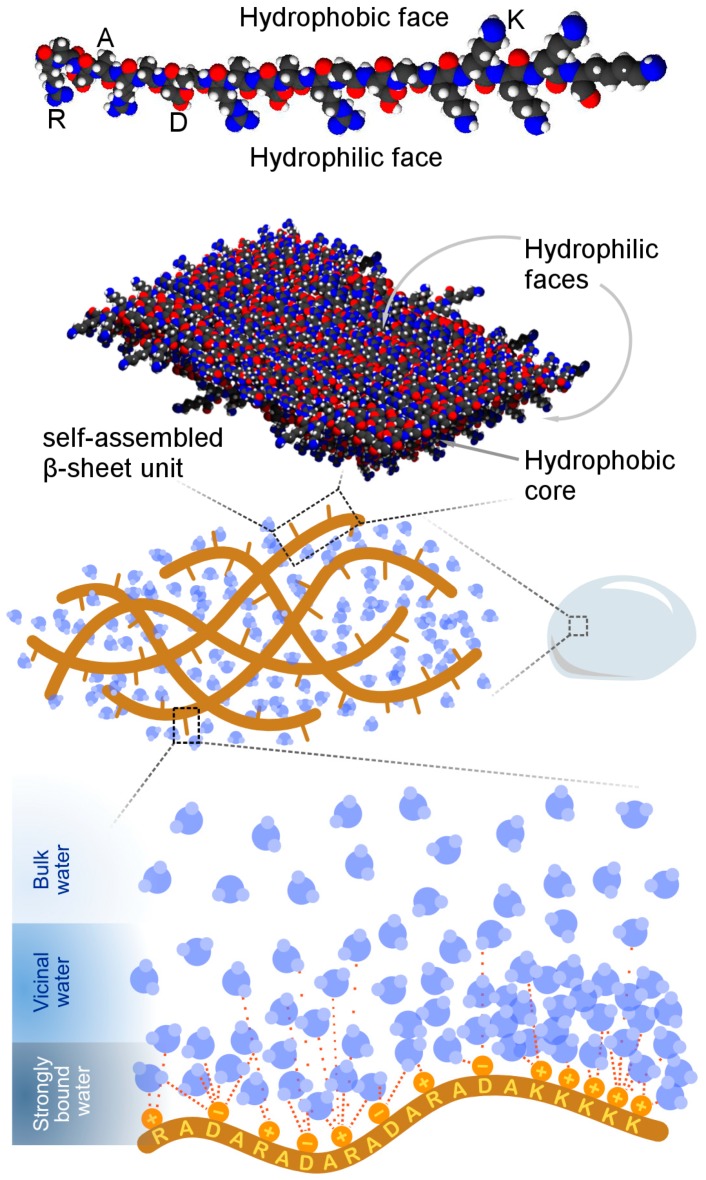
A working model of local water structure in lysinated (RADA)_4_ hydrogel. (RADA)_4_ self-assembles with (RADA)_4_K_5_ into β-sheets to form hydrated nanofibers. Water molecules exhibit varying degrees of interaction with the peptide nanofibers, ranging from minimal interaction (bulk water) to substantial intermolecular attraction (strongly bound water). Appending the charged lysine motif to (RADA)_4_ promotes water–peptide binding.

Taken together, the ^2^H NMR data present a more complex description of water dynamics in (RADA)_4_ hydrogels than the discrete freezing or non-freezing categorization of water as per DSC-based experiments, summarized in Figure 4. The NMR spectra show a gradual broadening into a powder pattern as the temperature is decreased, suggesting that water molecules are organized into a continuum of states within the hydrogel matrix. Such a distribution of environments ranges from the bulk to the strongly bound phases with intermediate levels of mobility and binding strength to the nanofiber. 

## 4. Conclusions

In this study, variable temperature ^2^H NMR was used to investigate the water structure within (RADA)_4_ hydrogels as a function of peptide concentration (1 and 5%) and the presence of charged lysine moieties. Increasing the peptide concentration may enlarge the surface area available for nanofiber–water interactions. The presence of charged groups could further enhance the binding of water. The earlier data from DSC corroborate with those of NMR, and NMR further differentiates the phases of water that closely interact with the peptide. The results presented herein give insight on the internal water environment within a self-assembled structure.
